# The impact of hostile abortion legislation on the United States maternal mortality crisis: a call for increased abortion education

**DOI:** 10.3389/fpubh.2023.1291668

**Published:** 2023-12-05

**Authors:** Anna Kheyfets, Shubhecchha Dhaurali, Paige Feyock, Farinaz Khan, April Lockley, Brenna Miller, Lauren Cohen, Eimaan Anwar, Ndidiamaka Amutah-Onukagha

**Affiliations:** ^1^Tufts University School of Medicine, Boston, MA, United States; ^2^Center for Black Maternal Health and Reproductive Justice, Tufts University School of Medicine, Boston, MA, United States; ^3^University of Michigan School of Medicine, Ann Arbor, MI, United States; ^4^Collective Energy for Nurturing Training in Reproductive and Sexual Health (CENTRS Health), Albuquerque, NM, United States

**Keywords:** abortion, health policy, abortion restrictions, maternal health, abortion education, racial disparities

## Abstract

The growing restrictive abortion policies nationwide and the Supreme Court decision on *Dobbs v. Jackson Women’s Health Organization* place increasing barriers to abortion access in the United States. These restrictions disproportionately affect low-income people of color, immigrants, and non-English speakers, and have the potential to exacerbate already existing racial inequities in maternal and neonatal outcomes. The United States is facing a Black maternal health crisis where Black birthing people are more than twice as likely to experience maternal mortality and severe maternal morbidity compared to White birthing people. Restrictions creating geographic, transportation, and financial barriers to obtaining an abortion can result in increased rates of maternal death and adverse outcomes across all groups but especially among Black birthing people. Restrictive abortion laws in certain states will decrease already limited training opportunities in abortion care for medical professionals, despite the existing abortion provider shortage. There is an immediate need for federal legislation codifying broad abortion care access into law and expanding access to abortion training across medical education. This commentary explores the impact of restrictive abortion laws on the Black maternal health crisis through multiple pathways in a logic model. By identifying current barriers to abortion education in medical school and residency, we created a list of action items to expand abortion education and access.

## Introduction

In 2021, over 90 restrictive abortion policies had been enacted in the United States (US); more than any other year on record since the *Roe v. Wade* Supreme Court ruling in 1973 (1). The *Roe v. Wade* decision reduced maternal mortality rates by 30–40% for people of color by securing access to safe and legal abortions (2). The Supreme Court’s decision on *Dobbs v. Jackson Women’s Health Organization* has overturned the 50 years precedent set by *Roe v. Wade,* resulting in an immediate impact on abortion access (3). This decision overturned the rulings of *Roe v. Wade* and *Planned Parenthood v. Casey*, removing federal protection for abortion access and allowing states to regulate, limit, or ban abortion. As of September 2019, the majority of reproductive-age people living in the US live in abortion-hostile states (4). The Supreme Court’s decision to overturn *Roe v. Wade* in the *Dobbs v. Jackson Women’s Health Organization* decision has paved the way for 28 states with laws in place or proposed to ban abortion almost entirely through new legislation or preceding trigger laws that previously could not be enforced following the *Roe v. Wade* ruling (5–7).

Currently, 11.3 million individuals have to travel over an hour to reach the nearest abortion clinic (8). The repercussions of each clinic closing ripple out as more pregnant people seek services at a smaller number of centers, impacting not only the distance patients have to travel but also the congestion of each center, as they serve both local patients and patients from nearby states (9). A 25-mile increase in travel distance has been associated with a 5% reduction in abortions; as abortion clinics close, the remaining clinics experience an influx of patients that results in a decrease in abortions in their community (9). The increase of patients at facilities that provide abortions as other nearby facilities close negatively impacts the delivery of other care offered at reproductive health care clinics, such as preventative breast exams, mammograms, and pap smears (10).

Low-income and birthing people of color have increased rates of abortion compared to White and high-income birthing people (11). The abortion rate among White individuals in the US is 10 per 1,000, while it is 27.1 per 1,000 among Black individuals (12). Approximately 70% of pregnancies that were documented in 2014 were reported as unintended among Black people, while the rates were 57 and 42% among Hispanic and White people, respectively (13). Increased hostility toward accessing abortion creates an even more dangerous climate for Black people, who are already 2–4 times as likely to experience maternal mortality and morbidity than their White counterparts (14). Socioeconomic status, racial discrimination, and disproportionate access to health care, including more effective forms of contraception, are pivotal determinants in experiencing unintended pregnancies and similarly limit abortion access. Black people live in states with the most restrictive policies regarding abortion (15).

Hostile restrictions to abortion access coupled with the pre-existing Black maternal health crisis will result in increased rates of mortality and morbidity among Black birthing people. One study estimates a total abortion ban in the United States would result in an additional 140 maternal deaths annually (16). This would be a 21% increase in maternal death and a 33% increase for non-Hispanic Black individuals (16). One study estimated that the closure of abortion clinics and early gestational age limits increase maternal mortality by 6–15 and 38%, respectively. Worldwide, unsafe abortion results in the loss of 68,000 lives annually (17). Restrictions on legal and safe abortion can force individuals to resort to unsafe abortions performed by untrained individuals in unsafe settings, using methods that fail to meet healthcare standards (18).

**Figure 1:**
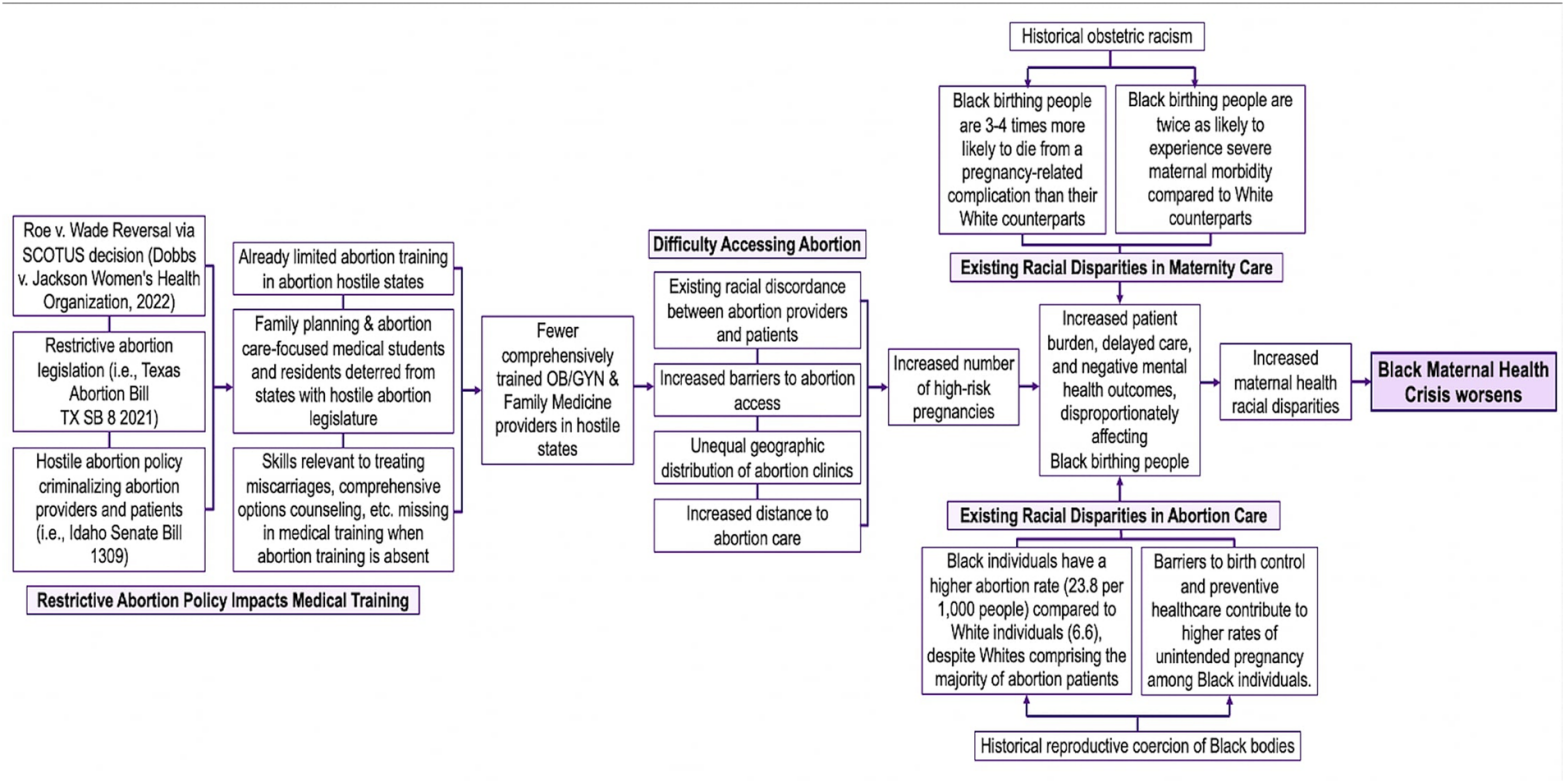
The impact of abortion restrictions on the US Black maternal health crisis logic model.

This commentary showcases the impact of restrictive abortion laws on the Black maternal health crisis through multiple pathways in a logic model. The logic model in Figure 1 explores the connections between abortion restrictions and the worsening Black maternal health crisis further, using abortion education and training as both a determinant and strategy (19–21).

### Looking forward: abortion education

Abortion education and training for medical students and residents, as well as related reproductive care, will become even more limited than it was prior to *Dobbs v. Jackson Women’s Health Organization* (22). These limitations on education will exacerbate racial inequities in maternal health by further limiting the quality of routine obstetric care in certain geographic regions that are already devastated by poor maternal health outcomes and by reducing opportunities to improve abortion provider diversity and provider concordance that was lacking prior to the Dobbs decision. In overturning *Roe v. Wade*, a distinction between essential healthcare and abortion has been made. However, routine obstetrical care includes abortion (23). It is imperative that future physicians have access to training on essential healthcare such as abortion. Similarly, abortion providers who have academic appointments in hostile states may be limited in what they can teach, and the number of clinical learning opportunities for abortion during the final 2 years of medical school will likely decrease (21, 22). The decision to overturn *Roe v. Wade* will not only make it more difficult for providers to perform abortions, but could also affect training in and care for patients requiring lifesaving miscarriage and ectopic pregnancy care (21, 24). Across various specialties, such as emergency medicine, residents find themselves weighing the options between facing criminal charges for performing an abortion, or losing their patient whose survival depends on access to an abortion (25). Lack of abortion training access will decrease the quality of care physicians provide and the quantity of physicians able to provide this care in abortion hostile states. Thus, we sought to explore the current atmosphere of abortion training and how it will impact the Black maternal health crisis in our logic model and narrative review.

## Abortion education in medical schools

By the age of 40, one in four American birthing people have undergone at least one abortion procedure in their lives, making abortion one of the most common healthcare procedures in the US (4, 26, 27). Professional organizations such as the American College of Obstetricians and Gynecologists (ACOG) recognize abortion as an important and core topic for medical education (28). Despite being one of the most widely utilized maternal health care services and recognized as an essential topic for medical education, the majority of US medical schools lack sufficient abortion education (27). While competing priorities and the breadth of information necessary to provide are causes of limitations in all preclinical education, one cause for the insufficient attention given to abortion during preclinical years lies includes the underlying sexism and racism present in medical education (29). Medical practice inadequately considers gender in the areas of diagnosis, treatment, and disease management for men, women, and gender minorities (30). Gender minorities have been systematically excluded from medical and scientific knowledge. As a consequence, the healthcare system has been shaped by and catered to men. This bias in healthcare and clinical research has far-reaching implications for obstetric health and medical practices compromising the quality of care provided to birthing persons (31, 32). The logic model in Figure 1 showcases the medical bias is worse for racial and ethnic minorities demonstrated by the current Black maternal mortality crisis rooted in the history of obstetric racism present in the US.

There is very limited data on abortion curricula in US medical schools (33). One of the few studies published on this topic demonstrated that abortion education is not thoroughly incorporated into medical schools’ curricula: 17% of medical schools in the US did not formally teach abortion, and less than 50% of schools dedicated at least one lecture on abortion (26). Of the schools that offered clinical abortion care experience, it was included in the third year of medical school as an elective course that interested students had to actively seek out (26). Another study requesting information from the 126 accredited US medical schools’ OB/GYN clerkship directors found that nearly a quarter of schools offered no formal abortion education in their clinical and preclinical program years, and a majority of schools only offered one abortion-care lecture elective course (34). An updated preliminary 2020 study reported that since 2005, there have been increases in abortion education availability in American medical schools, but compared to the national demand, the increases are insufficient (35). This is only set to progressively worsen with abortion education being limited in nearly half of the country.

In the year following the Dobbs decision (2022–2023), states with the most severe abortion restrictions found a 3.0% decrease in all applicants into residency programs, with a 10.5% decrease in OB/GYN applicants compared to previous application cycles (36). In a single application cycle, the impact of the Dobbs decision and subsequent abortion bans and restrictions has been made clear by these graduating medical students choosing to practice in other states. This change foreshadows a decrease in the number of physicians in states with abortion restriction, in OB/GYN as well as other specialties.

## Abortion training in residency

The Accreditation Council for Graduate Medical Education (ACGME) and ACOG require and recommend all 267 accredited obstetrics and gynecology residency programs in the US provide access to abortion training and routinely teach abortion care to their residents (33). A study published in 2019 surveying OB/GYN Program Directors found that out of 190 respondents, 10 programs do not offer any abortion training at all (5%), 59 offer optional abortion training (31%), and 121 programs routinely schedule training for their residents (64%) (37). This is concerning as contraception, miscarriage management, medication and surgical abortion methods are highly necessary and routine health procedures for a large part of the US population (4).

Recent years have demonstrated increased integration and abortion care training among family medicine physicians. Family medicine physicians are the most common specialty in medicine practicing in abortion-care deserts, places with a lack of abortion-care/abortion-care access limitations (38). In a nationally representative sample of family medicine physicians, over 80% described having treated early pregnancy loss and 73% agreed that abortion was within their scope of practice, whereas only about 15% of family medicine providers in this survey reported offering early abortion care. This discrepancy may be explained by the fact that only 7% of all nationally accredited family medicine residencies offer abortion-care training (38). All medical practitioners who serve reproductive-aged birthing people must understand and be able to adequately facilitate abortion care and comprehensive family planning counseling, even if they do not perform the abortions themselves (33).

Following the Dobbs decision overturning *Roe v. Wade*, approximately 44% of residents in OB/GYN programs will no longer have access to in-state abortion training (39). Before Dobbs, residents in Missouri had to go to Illinois to be fully trained in abortion, now traveling elsewhere to practice these skills will become a reality for residents in Texas and other states that are hostile to abortion, though coordinating this effort will be difficult (21). Physicians in Louisiana are concerned that they will not be able to recruit the best physicians to the state due to the new laws limiting abortion training and provision opportunities, impacting the quality of care for its residents (24).

## Barriers for providers

Over the past several years, the number of abortion providers in most states has significantly declined. As of 2017, 89% of all US counties do not have an abortion provider available for their residents (4). The abortion provider decline is associated with the increasingly restrictive and hostile abortion legislation taking hold in the US (4, 40). Over the last decade, there have been 479 abortion restrictions enacted in 33 states, even though abortion is one of the safest medical procedures (40).

States with abortion bans or restrictions experience adverse outcomes including limited maternity care providers, maternity care deserts, higher rates of maternal mortality and infant death, especially among people of color, elevated death rates for birthing individuals of reproductive age, and greater racial disparities in healthcare (41, 42). Maternal death rates in abortion-restriction states were 62% higher than in states with greater abortion access states (28.8 vs. 17.8 per 100,000 births) (43). Abortion-restrictive states have a 32% lower ratio of obstetricians to births and a 59% lower ratio of certified nurse midwives to births compared to states with abortion access (41). The recent Dobbs decision could exacerbate this disparity as it may deter some maternity care providers from practicing in states where their work faces legal challenges, as seen in the recent residency application cycle (36). Insufficient maternity resources not only restrict access to birthing services, but also make it harder for pregnant individuals to access early and continuous prenatal care. In 2020, states with abortion restrictions had a 62% higher proportion of individuals giving birth who either received no prenatal care or received it late when compared to states with abortion access (44).

Surveyed Maternal-Fetal Medicine (MFM) providers stated that individual, institutional, and state-level factors impact their ability to provide abortion care in their practices (40). Limitations such as abortion public funding, cost, state mandates, waiting periods, and institutional policies impact their ability to provide abortion care (40). MFM physicians practicing in supportive abortion legislation states reported higher abortion provisions than those physicians practicing in abortion-hostile states, resulting in an unequal geographic distribution and representation of abortion providers and abortion clinics across the US and reduced access to reproductive health services (40). The disproportionate distribution of physicians is especially dangerous for high-risk patients whose pregnancies pose impending physical threats to their lives and who are located in areas with reduced or no access to family planning counseling services (Figure 1). All these factors readily contribute to the rising US maternal mortality rates, especially for Black birthing people who face more deadly birth inequities that are slated to worsen as states further eliminate access and support for abortion (15, 40). Abortion providers and clinicians standing up to these injustices are facing immense backlash. For example, a physician in Indiana publicly shared a story of her 10-year-old patient who was raped and could not obtain an abortion in their home state; subsequently she was humiliated by state attorneys, called a liar, and is now facing legal troubles (45).

## Provider concordance

Abortion hostility and restrictive legislation throughout institutions is not the only problem in accessing abortion and reproductive health care services, or training abortion provider. The abortion provider and abortion care workforce does not reflect the communities it serves. After centuries of canceled and compromised reproductive autonomy, Black birthing people once again find their health and rights in the hands of people who do not share their lived experiences. The majority of abortion care providers are White and serve largely non-White, immigrant, low-income, and non-English speaking populations (46, 47). This is a result of the systematic exclusion of people of color from the medical profession and results in the exclusion and stigmatization of patients (48). Nearly half of all abortions obtained in the US are by those whose incomes are below the federal poverty level (46). Despite this, wealthy, White individuals still hold the greatest power and leverage over the legislative decisions being made, the pathways created for education, pathways for employment and work, and education curricula surrounding abortion and reproductive health care. As training opportunities for abortion care become more limited across the country, there is further limitation to training culturally concordant providers.

Diverse physicians, healthcare specialists, and administrators are associated with improved health outcomes for underserved, vulnerable, underrepresented, and underprivileged patient populations (49). Not only are there improved health outcomes but a more diverse physician workforce is also associated with White doctors being more culturally competent and better serving minority patients (50). There must be increased workforce diversity in the physician and medical care workforce as a whole, and in abortion provision in particular, as cultural humility, competence, and respect are essential in creating an unbiased, quality healthcare system rooted in justice and equity (51). As opportunities for training become more limited with the elimination and severe restriction of abortion access, increasing provider concordance will become even more difficult, and should remain a focus of programs seeking to improve health equity.

## Call to action

In recent years, with advocacy efforts from Medical Students for Choice, the Kenneth J. Ryan Program, and Reproductive Health Education in Family Medicine (RHEDI) programs, the availability of abortion education in some US medical schools has improved (4, 27, 52). The overturn of *Roe v. Wade* will undoubtedly impose limits on education related to miscarriages and other OBGYN health issues (21). To combat this, abortion education must be embedded into the overall medical school curriculum for all US medical schools (27). The healthcare field should be intentional in training the next generation of clinicians. This can be accomplished by requirements set forth by the American Medical Association, Association of American Medical Colleges, and the American Association of Colleges of Osteopathic Medicine, for all medical schools to include evidence-based abortion education in their preclinical curricula, and as possible in their clinical years. For schools in states with limited training to abortion, efforts should be made to offer abortion training experiences or dedicated time to establish them in other states during clinical years. Further, standardized exams can demonstrate the ubiquity of and normalize abortion by including the topic as an unstigmatized procedure on the United States Medical Licensing Exams and Comprehensive Osteopathic Medical Licensing Examinations. It is crucial to incorporate abortion training into the medical school curriculum, similar to any other surgical or medical procedure, to diminish its associated stigma (28).

Both residents and medical students should be supported by their respective institutions for advocacy work being done to improve access to abortion care. Residents in specialties adjacent to abortion care including pediatrics, anesthesia, and emergency medicine, should be trained on counseling for abortion care options and where to refer patients. Programs that offer abortion training must also be intentional in recruitment of trainees. Not only should the number of abortion providers in training increase, but also the racial concordance between physician and patient should be considered as a determinant of patient experience and outcomes.

Attention should be focused on improving access to abortion medication outside the clinic setting. Self-managed abortions are as safe as those in the clinic and online telemedicine can be highly effective (53, 54). Most importantly, physicians of any specialty should not report individuals who seek care following a self-managed abortion. Legislative action is necessary to secure reproductive rights long-term. The healthcare field should advocate for establishing federal law securing access, in particular, to abortion and reproductive healthcare, including federally enacting the Women’s Health Protection Act (55). Given the fact that nearly one-quarter of birthing people in the US will have an abortion in their lifetimes and that abortion restrictions disproportionately impact already vulnerable populations, the medical community must leverage its power to protect the right to abortion and provide appropriate resources through advocacy.

## Data availability statement

The original contributions presented in the study are included in the article/supplementary material, further inquiries can be directed to the corresponding author.

## Author contributions

AK: Conceptualization, Data curation, Investigation, Methodology, Project administration, Visualization, Writing – original draft, Writing – review & editing. SD: Conceptualization, Data curation, Investigation, Visualization, Writing – original draft, Writing – review & editing. PF: Conceptualization, Data curation, Investigation, Writing – original draft, Writing – review & editing. FK: Conceptualization, Data curation, Supervision, Writing – review & editing. AL: Conceptualization, Data curation, Supervision, Writing – review & editing. BM: Investigation, Writing – original draft. LC: Writing – original draft, Writing – review & editing. EA: Writing – original draft, Writing – review & editing. NA-O: Conceptualization, Supervision, Writing – review & editing.
