# Corrigendum: The impact of hostile abortion legislation on the United States maternal mortality crisis: a call for increased abortion education

**DOI:** 10.3389/fpubh.2024.1358617

**Published:** 2024-02-02

**Authors:** Anna Kheyfets, Shubhecchha Dhaurali, Paige Feyock, Farinaz Khan, April Lockley, Brenna Miller, Lauren Cohen, Eimaan Anwar, Ndidiamaka Amutah-Onukagha

**Affiliations:** ^1^Tufts University School of Medicine, Boston, MA, United States; ^2^Center for Black Maternal Health and Reproductive Justice, Tufts University School of Medicine, Boston, MA, United States; ^3^University of Michigan School of Medicine, Ann Arbor, MI, United States; ^4^Collective Energy for Nurturing Training in Reproductive and Sexual Health (CENTRS Health), Albuquerque, NM, United States

**Keywords:** abortion, health policy, abortion restrictions, maternal health, abortion education, racial disparities

In the published article, there was an error in the author list, and authors Lauren Cohen and Eimaan Anwar were erroneously excluded. The corrected author list appears below.

Anna Kheyfets^1,2*^, Shubhecchha Dhaurali^2^, Paige Feyock^2,3^, Farinaz Khan^4^, April Lockley^4^, Brenna Miller^2^, Lauren Cohen^2^, Eimaan Anwar^2^ and Ndidiamaka Amutah-Onukagha^1,2^

The Author Contributions statement has been updated accordingly, with “Writing – original draft, Writing – review & editing” added for Lauren Cohen and Eimaan Anwar.

The authors apologize for this error and state that this does not change the scientific conclusions of the article in any way. The original article has been updated.

